# Serum procalcitonin as a marker of neonatal intrahepatic cholestasis caused by citrin deficiency (NICCD)

**DOI:** 10.1016/j.clinsp.2024.100383

**Published:** 2024-05-25

**Authors:** Tao Jiang, Wen-Xian Ouyang, Yan-Fang Tan, Ying Yu, Xiao-Mei Qin, Hai-Yan Luo, Lian Tang, Hui Zhang, Shuang-Jie Li

**Affiliations:** a Department of Hepatopathy Center, Hunan Children's Hospital, Changsha, China; b The first Department of Emergency General, Hunan Children's Hospital, Changsha, China

**Keywords:** Diagnosis, Prognosis, Neonatal intrahepatic cholestasis, Citrin deficiency

## Abstract

**Background:**

Neonatal Intrahepatic Cholestasis (NICCD), as the early-age stage of Citrin deficiency involving liver dysfunction, lacks efficient diagnostic markers. Procalcitonin (PCT) has been identified as a biomarker for infection as well as various organ damage. This study aimed to explore the potential of PCT as a biomarker for NICCD.

**Methods:**

In a single-center retrospective case-control study. Serum PCT concentrations before and after treatment of 120 NICCD patients, as the study group, were compared to the same number of cholestatic hepatitis patients, as the control group. The potential value of PCT to discriminate NICCD from control disease was further explored using Receiver Operating Characteristic (ROC) curve analysis and compared to those of other inflammatory markers.

**Results:**

There was a significantly higher level of PCT in NICCD patients than in the control group. PCT concentrations were only weakly correlated with neutrophil counts and CRP levels (p ˂ 0.05). At a cut-off value of 0.495 ng/mL, PCT exhibited a significantly higher diagnostic value compared to other inflammatory markers for discriminating NICCD from the control, with a sensitivity of 90.8 % and specificity of 98.3 %.

**Conclusion:**

PCT might be used as an initial biomarker to discriminate children with NICCD from another hepatitis disease.

## Highlights

• PCT was a highly sensitive marker to differentiate NICCD from cholestatic hepatitis patients.
• PCT was reduced when NICCD was treated.
• PCT is a much more effective marker to differentiate NICCD than the inflammatory markers.

## Introduction

Citrin deficiency is a recessively inherited metabolic disorder. Functioning as an Aspartate Glutamate Carrier (AGC) expressed mainly in the liver, kidney, and heart, Citrin deficiency may result in a disturbance in urea synthesis and malate-aspartate NADH shuttle, which can lead to various metabolic abnormalities.^1^ Citrin deficiency exhibits several age-dependent clinical manifestations: in newborn and infant stages, it could cause neonatal cholestasis, defined as conjugated hyperbilirubinemia in the newborn period or shortly thereafter, therefore referred to as Neonatal Intrahepatic Cholestasis (NICCD: OMIM 605,814); in older children, deficiency of citrin could exhibit as Failure to Thrive and Dyslipidemia (FTTDCD); in adults, it causes type II Citrullinemia (CTLN2: OMIM 603,471).^2^ NICCD was mostly self-resolving with treatments mainly aiming at preventing the progression of cholestasis. Then there usually will be an adaptation/compensation and asymptomatic stage. However, adult-onset CTLN2 may present with hyperammonemia, liver steatosis, and neuropsychiatric symptoms, even leading to death.^1^ Therefore, timely diagnosis and management of Citrin deficiency is of great importance.

The diagnosis of Citrin deficiency could be initiated from clinical observation such as intrahepatic cholestasis, as well as different biochemical findings (such as plasma ammonia, citrulline, various amino acids, and pancreatic secretory trypsin inhibitor). However, the confirmatory diagnosis has to be dependent on genetic analysis of mutant SLC25A13.^3^ Missed diagnoses^4^, ^5^, ^6^ and false negative results^7^ have been often noticed since NICCD symptoms were mostly transient. Therefore, it was very important to find reliable diagnostic indicators.

Procalcitonin (PCT) is a 116-amino acid residue produced in the parafollicular (C cells) of the thyroid gland. It is then converted to calcitonin, a 32-amino acid hormone involved in serum calcium regulation. PCT is a very stable protein in vivo and in vitro, making it feasible as a biomarker in various clinical conditions. In normal conditions, the serum contains negligible procalcitonin.^8^^,^^9^ Microbial toxins such as endotoxin and lipopolysaccharide and inflammatory factors can stimulate the synthesis of PCT in multiple tissues, including the liver, kidney, adipocytes, pancreas, colon, and brain.^10^^,^^11^ The elevation of PCT levels seems to be specific for bacterial infection while virus infection can reduce PCT, therefore it has been utilized as a sensitive biomarker to evaluate infection severity and to facilitate instruction of antibiotic usage.^12^^,^^13^ In recent years, it has been suggested that PCT serum levels may also be elevated in various noninfectious conditions including chronic obstructive pulmonary disease,^14^ cardiovascular diseases,^15^ kidney injury.^16^ In addition, serum PCT levels have also been reported to be correlated with metabolic disorders.^17^, ^18^, ^19^ Previous studies have also suggested that PCT levels were associated with liver disease,^20^^,^^21^ especially those accompanied by infection.^22^

Considering that changes in PCT levels may simultaneously reflect infection, metabolic disorder, as well as liver organ damage, it might be a useful biomarker to facilitate the identification of NICCD. In the study reported here, the authors explored the potential usefulness of PCT to discriminate NICCD from cholestatic hepatitis.

## Materials and methods

### Study design

In a single-center retrospective case-control study, patients diagnosed with Citrin deficiency (NICCD, study group) and cholestatic hepatitis (as control) between January 2014 to October 2019 at the Department of Hepatopathy Center of Hunan Children's Hospital in Changsha (Hunan Province, China) were reviewed. PCT levels in serum samples were measured and compared between the two groups. The diagnostic value of PCT to discriminate NICCD from cholestatic hepatitis was determined using Receiver Operating Characteristic (ROC) curve analysis. The study was approved by the Ethics Committee at Hunan Children's Hospital (KS2021–59), and informed consent was obtained from each patient. This study follows the STROBE Statement.

### Participants

Children diagnosed with Citrin deficiency (NICCD) children were identified through clinical record review. Inclusion criteria were no fever or bacterial infection in the records; had available blood samples reserved before and after treatment. As controls, patients diagnosed with cholestatic hepatitis due to other pathology at the same period, with reserved blood samples available, were randomly selected.

### Variables data extraction

Citrin deficiency diagnosis was made based on the clinical manifestations and genetic analysis of SLC25A13 mutations.^23^ The diagnosis was reconfirmed according to clinical records during data extraction.

Other extracted baseline data at diagnosis included demographics (gender, age, height, weight), and blood and laboratory test results (white blood cell count, hemoglobin, liver and renal function, myocardial enzyme, electrolyte, glucose, lipids, lactic acid, ammonia and coagulation factors).

Treatments of NICCD patients were also extracted from the records. All diagnosed NICCD were fed with Medium Chain Triglycerides (MCT) milk and lactose-free milk and received treatment with Diisopropylamino Dichloroacetate (20 mg ivgtt qd, 5‒7d), Ursodeoxycholic acid (10‒20 mg/kg qd 5‒7d) and probiotics.

### PCT measurement

The PCT level was determined using electrochemiluminescence (reagent: Elecsys BRAHMS PCT, instrument: electrochemiluminescence automatic immune analyzer, (cobas e 411, Japan). A value of 0∼0.05 ng/mL was considered as normal.

### Statistical analysis

All statistical analyses were performed using the SPSS Statistics software (version 23.0, IBM, Armonk, NY, USA) and R software (version 4.2.3). The normality of continuous variables was assessed using the Kolmogorov-Smirnov test. Normally distributed data were presented as mean ± standard deviation and differences between groups were compared using *t*-tests. Skewed data are presented as median (25th to 75th percentiles), and differences between groups were compared using the Mann-Whitney *U* test. Categorical data are presented as numbers (percentages), with differences between groups compared using Chi-Square, or Fisher's exact test when one or more cells had an expected frequency of five or less. Due to the skewed distribution of data, a paired Wilcoxon signed-rank test was used to compare PCT levels pre- and post- treatment. The linear regression model of Spearman's correlation analysis was used to determine the association between PCT and other test parameters. Receiver Operating Characteristic (ROC) curve analysis was employed to determine the potential diagnostic value of PCT to discriminate NICCD from cholestatic hepatitis. The cutoff was determined by calculating the highest Youden index (sensitivity+specificity^−1^). Statistical significance was determined by a p-value < 0.05.

## Results

### Participants

The 120 NICCD patients included were identified with seven mutations: (I) 851del4; (II) IVS11 +1G>*A*; (III) 1638ins23; (IV) S225X; (V) IVS13 +1G>*A*; (VIII) E601X; and (XIX) IVS16ins3kb. Combinations of these mutations yielded the following 11 genotypes: I/I (*n* = 4), I/II (*n* = 2), I/IV (*n* = 2), I/XIX (*n* = 1), II/II (*n* = 1), II/IV (*n* = 2), II/V (*n* = 3), II/VIII (*n* = 1), III/XIX (*n* = 1), IV/XIX (*n* = 2), and V/V (*n* = 1), respectively. The allele frequencies were I (32.5 %), II (25.0 %), III (2.5 %), IV (15.0 %), V (12.5 %), VIII (2.5 %), and XIX (10.0 %). Baseline characteristics are presented in Table 1. The majority (108) of the children were aged 1‒4 months and the other 12 were aged 4‒7 months. There were 75 males and 45 females. Three of them had liver cirrhosis and one case had liver failure.

**Table 1:** Characteristics and biochemical indices.

Variables	NICCD (*n* = 120)	Control (*n* = 120)	p-value	Z/χ2
Age: (year), Median (P25, P75)	2 (1.71, 2.72)	1.89 (1.28, 2.20)	0.0100^a^	3.40
Gender:(% male)	75 (62.5 %)	79 (65.8 %)	0.6861^b^	0.29
Clinical patterns:				
Jaundice; n (%)	100 %	100 %	‒	‒
Clay-colored stools n (%)	7 (5.83 %)	3 (2.5 %)	0.1964^b^	1.67
Diarrhea; n (%)	10 (8.33 %)	4 (3.33 %)	0.0980^b^	2.73
Selected clinical parameters				
Wbc(10^^9^/L) Median (P25, P75)	12.58 (10.31, 15.28)	10.70 (9.01, 12.66)	0.0000^c^	−4.453
Leukocytes (10^^9^/L) Median (P25, P75)	7.02 (5.21, 9.15)	6.76 (5.38, 8.71)	0.6330 c	−4.77
Neutrophils (10^9/L) Median (P25, P75)	3.81 (2.69, 5.09)	2.38 (1.77, 2.95)	0.0000^c^	−7.891
Hemoglobin (g/L) Median (P25, P75)	100 (88, 109)	102 (94, 116)	0.0318^c^	−2.151
Platelet(10^^9^/L) Median (P25, P75)	466 (381, 603.25)	412(338, 529)	0.0038^c^	−3.016
C reactive protein (mg/L), Median (P25, P75)	2.05 (0.63, 3.56)	0.57 (0.5, 1.4)	0.0000^c^	−5.529
Total bilirubin (μmoL/L), Median (P25, P75)	171.75 (122.61, 217.29)	135.4 (87.95,174.5)	0.0000^c^	−4.309
Conjugated bilirubin (μmoL/L), Median (P25, P75)	73.87 (54.59, 98.59)	86.65 (58.45, 120.28)	0.0567^c^	−1.192
Indirect bilirubin (μmoL/L), Median (P25, P75)	90.82 (56.38, 120.14)	37.55 (25.23, 57.18)	0.0000^c^	−8.282
Albumin (g/L), Median (P25, P75)	30.14 (27.28, 33.37)	37.5 (35.63, 39.6)	0.0000^c^	−11.002
Globin (g/L), Median (P25, P75)	15.8 (13.53, 18.05)	17.15 (15.1, 19.28)	0.0000^c^	−3.543
Glutamic-pyruvic transaminase (IU/L), Median (P25, P75)	42.05 (31.3, 56.55)	103.6 (64.25, 170.75)	0.0000^c^	−8.929
Glutamic-Oxaloacetic transaminase (IU/L), Median (P25, P75)	96.63 (71.27, 134.98)	132.45 (90.13, 202.18)	0.0000^c^	−3.656
Total bile acid (IU/L), Median (P25, P75)	174.05 (125.79, 208.38)	106.4 (82.7, 136.15)	0.0000^c^	−7.770
Gamma-glutamyl transferase (μmoL/L), Median (P25, P75)	176 (145.39,225.85)	124 (85, 221.05)	0.0000^c^	−3.586
Glycerin dilaurate (mmoL/L), Median (P25, P75)	1.48 (1.2, 2)	1.63 (1.01, 2.09)	0.9932^c^	−0.009
Cholesterol (mmoL/L), Median (P25, P75)	4.34 (3.59, 5.13)	3.7 (3.02, 4.41)	0.0000^c^	−4.109
Blood sugar(mmoL/L), Median (P25, P75)	4.12 (2.92, 5.02)	4.64 (3.72, 5.13)	0.0065^c^	−2.767
Blood lactic acid(mmoL/L), Median (P25, P75)	4.43 (3.14, 5.48)	3.73 (2.83, 4.6)	0.0010^c^	−3.383
Blood ammonia (μmoL/L), Median (P25, P75)	81.2 (51, 103)	54.3 (39.6, 75.93)	0.0000^c^	−5.237
Prothrombin time(*sec*), Median (P25, P75)	16.15 (14.73, 18.68)	12.9 (12.3, 13.5)	0.0000^c^	−11.530
International normalized ratio (INR), Median (P25, P75)	1.3 (1.16, 1.56)	0.98 (0.93, 1.04)	0.0000^c^	−11.522
APTT (*sec*), Median (P25, P75)	47.85 (42.98, 54)	43.6 (40.5, 46.78)	0.0000^c^	−5.332
Plasma fibrinogen (mg/dL), Median (P25, P75)	119.75 (100, 153)	284 (254.5, 337.25)	0.0000^c^	−12.427
PCT (ng/ml), Median (P25, P75)	1.51 (0.88, 2.40)	0.21 (0.14, 0.30)	0.0000^c^	−12.573

WBC, White Blood Cells; APTT, Activated Partial Thromboplastin Time.

a Mann-Whitney *U* test.

b Chi-Square test.

c Mann-Whitney *U* test.

The same number of control patients were included. Comparisons of baseline characteristics between the two groups of patients found statistically significant differences in various blood test parameters. Noticeable larger differences were noticed in indirect bilirubin, glutamic-pyruvic transaminase, and plasma fibrinogen, along with explicit differences in neutrophils, CRP, bile acid, Gamma-glutamyl transferase, lactic acid, and ammonia. The other differences appeared to be small, although with statistical significance. These results indicated that the patients in the two disease groups might had different levels of liver damage, metabolism, as well as inflammation.

### Serum PCT levels in NICCD and control groups

As shown in Table 1, the median PCT level in the NICCD group (1.51 ng/mL) was significantly (p ˂ 0.0001) higher than that in the control group (0.26 ng/mL). In addition, In 69 patients with both pre- and post-treatment blood samples were available. Significant decreases of serum PCT levels after treatment were detected compared to pre-treatment (Table 2). These results indicated a significant correlation between serum PCT level with NICCD.

**Table 2:** Comparison between pre- and post-treatment PCT levels.

Vatable	Pre-treatment	Post-treatment	p-value	Z-value
Procalcitonin(ng/mL), Median (P25, P75)	1.77 (1.23, 2.82)	0.37 (0.25, 0.72)	0.0000^a^	−7.323

a Paired Wilcoxon signed-rank test.

### Diagnostic value of PCT to discriminate NICCD from cholestatic hepatitis

**Fig. 1:**
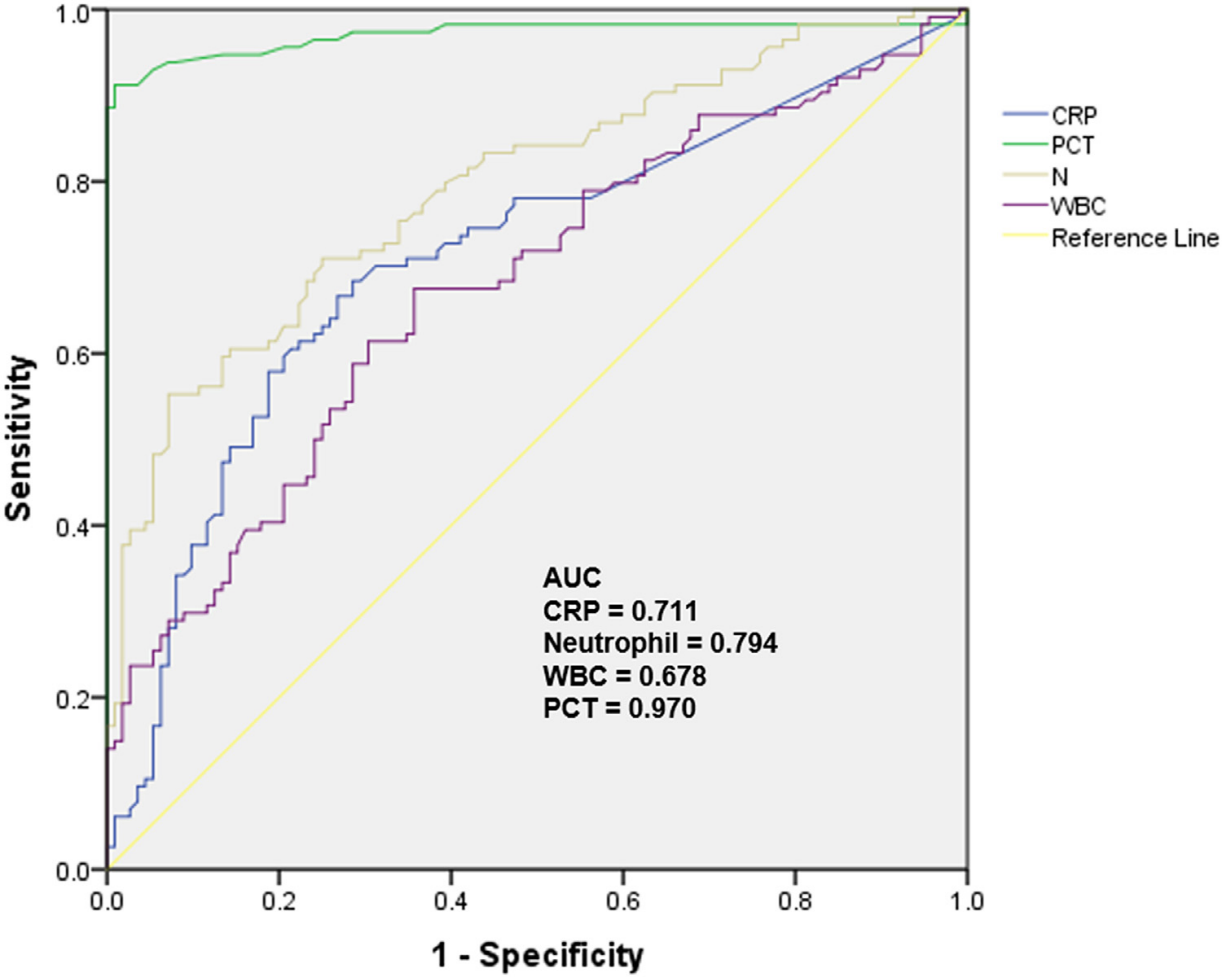
Receiver-Operating Characteristic (ROC) curve analysis of diagnostic value of serum PCT level and inflammatory markers for differentiating NICCD from cholestatic hepatitis.

Results of ROC analysis (Fig. 1) showed that PCT had significant diagnostic value for NICCD, with AUC = 0.968 (95 % CI 0.944, 0.996), sensitivity = 90.8 %, specificity = 98.3 %, and a cut-off of 0.495 ng/mL. On the other hand, inflammatory markers CRP, neutrophil, and WBC, although also reached significances for diagnostic value, were significantly less effective, with AUC of 0.711 (95 % CI 0.643, 0.780), 0.794 (95 % CI 0.736, 0.862), and 0.678 (95 % CI 0.609, 0.748), respectively.

### Correlation between PCT and other serum markers

To explore the potential mechanism that connects NICCD to PCT elevation. The authors tested the correlation between PCT with various serum markers of inflammation, organ functions, metabolism and coagulation. For primary exploration of the potential correlation between these quantitative data, a linear Spearman model analysis was used. As shown in Table 3, the results showed that PCT had only a weakly positive correlation with neutrophils (*r* = 0.223), CRP (*r* = 0.229), and conjugated bilirubin (*r* = 0.191) but no correlation with other variables. These results indicated that the changes in PCT in NICCD may not be explained by a single mechanism such as infection, organ damage, or metallic disorder, but rather reflect a combination of multiple mechanisms.

**Table 3:** Correlation analysis between serum PCT levels and other serum markers in NICCD patients.

Biomarkers	*r* value	p-value
WBC (10^9/L)	0.098	0.2896
Leukocytes (10^9/L)	0.034	0.7134
Neutrophils (10^9/L)	0.212	0.0202*
Hemoglobin (g/L)	−0.098	0.2888
Platelet (10^9)	0.118	0.1981
C reactive protein (mg/L)	0.248	0.0064*
Total bilirubin (μmoL/L)	0.143	0.1182
Conjugated bilirubin (μmoL/L)	0.191	0.0362*
Indirect bilirubin (μmoL/L)	0.09	0.3273
Albumin (g/L)	0.01	0.9189
Globin (g/L)	−0.64	0.4888
Glutamic-pyruvic transaminase (IU/L)	−0.065	0.4797
Glutamic-oxaloacetic transaminase (IU/L)	−0.112	0.2253
Total bile acid (IU/L)	−0.08	0.3853
Gamma-glutamyl transferase (μmoL/L)	−0.048	0.6068
Glycerin dilaurate (mmoL/L)	−0.038	0.6786
Cholesterol (mmoL/L)	−0.09	0.3284
Blood sugar (mmoL/L)	−0.085	0.3573
Blood lactic acid (mmoL/L)	−0.005	0.9532
bBlood ammonia (μmoL/L)	−0.126	0.1708
Prothrombin time (*sec*)	0.063	0.4965
International normalized ratio (INR)	0.053	0.5676
APTT (*sec*)	−0.071	0.4391
Plasma fibrinogen (mg/dL)	−0.083	0.3675

WBC, White Blood Cells; APTT, Activated Partial Thromboplastin Time.

## Discussion

PCT as a biochemical marker has been widely explored in the diagnosis and prognosis of various diseases. To our knowledge, this is the first study to examine the relationship between PCT and the NICCD. The results showed that PCT was a highly sensitive marker to differentiate NICCD from cholestatic hepatitis patients and that PCT was reduced when NICCD was treated.

In this study, the authors found that a cut-off value of 0.495 ng/mL performed well (with a sensitivity of 90.8 % and specificity of 98.3 %) in the diagnosis of NICCD. Although PCT is a widely used biomarker for the diagnosis of bacterial infections. Studies did not show such high sensitivity and specificity in the diagnosis of liver disease with bacterial infection. The previous study^22^ has found that when used for sepsis from alcoholic hepatitis, at a cut-off value of 0.57 ng/mL, PCT had a sensitivity of 79 % and a specificity of 82 %. Moreover, when used as a predictor of spontaneous bacterial peritonitis SBP, at a cut-off value of > 0.78 ng/mL, was with only a sensitivity of 77.5 % and specificity of 60.4 %. These results indicated that PCT can reflect liver dysfunction in Citrin deficiency with a very high specificity.

The NICCD patients in this study were with no concomitant infection records. Although the inflammatory markers indicate that there might be infections in both NICCD and control patients, it may also be from non-infection causes. Liver injury results in the death of a vast amount of liver cells and the release of Damage-Associated Molecular Patterns (DAMPs), which cause systemic inflammation or other changes in immune responses,^24^^,^^25^ including the production of various inflammatory mediators (e.g., the Tumor Necrosis Factor [TNF] and Interleukin [IL]−1β, IL-6, IL-8, IL-10).^26^^,^^27^ It has been suggested that systemic inflammation and significant increases in IL-1β and TNF-a can induce the expression of PCT.^28^^,^^29^ Therefore, the correlations between PCT and levels of different inflammatory factors were explored. Only weak positive correlations between PCT with neutrophils and CRP were found in NICCD patients from this study. In addition, the PCT levels showed no clear trend of changes in 3 patients with cirrhosis and 1 patient with liver failure (2.3 ng/mL, 0.37 ng/mL, 1.57 ng/mL, and 0.75 ng/mL respectively). Thus, the observed changes in PCT levels in NICCD patients could not be simply explained by systematic inflammation or metabolism changes. The comparisons among ROC curves also indicated that PCT is a much more effective marker to differentiate NICCD than the inflammatory markers.

PCT was originally identified and considered as a biomarker for bacteria but not viral infection.^30^ The exact cause that induces PCR release remains to be elusive. The biomarker has been suggested to be indicative in different organ damage detection,^14^^,^^15^^,^^21^ while not a simple reflection of inflammatory injury.^30^ NICCD, or Citrin deficiency is a metabolic disorder that mainly causes disturbance in urea synthesis and malate-aspartate NADH shuttle function, which may result in a disorder in energy metabolism.^1^^,^^31^ Whether the specificity of PCT in detecting NICCD is an indicator of organ damage, or it may also reflect metabolic changes, will be an interesting question for further analysis.

The study is subjected to certain limitations. This was a single-center study with a small sample size. Therefore, the interpretation of the results in this study is limited. Studies with larger sizes and more diverse samples are needed in the future to verify the reliability of our conclusions.

## Conclusions

The serum concentration of PCT was increased in Citrin deficiency patients. PCT greater than 0.495 ng/mL may be used to initially differentiate NICCD from cholestatic hepatitis. The levels of PCT decreased significantly after treatment. Further research is warranted in the future to identify the procalcitonin levels distinguishing NICCD from various other types of hepatic cholestasis.

This work was supported by the 2019 National Medical Service and Support Capacity Improvement Project (Medical and Health Institution Capacity Building), and the Clinical Research (Translation) Center of Hunan Children's Hospital.

## CRediT authorship contribution statement

**Tao Jiang:** Conceptualization, Writing – original draft, Data curation. **Wen-Xian Ouyang:** Writing – review & editing. **Yan-Fang Tan:** Data curation. **Ying Yu:** Resources. **Xiao-Mei Qin:** Resources. **Hai-Yan Luo:** Writing – review & editing. **Lian Tang:** Resources. **Hui Zhang:** Resources. **Shuang-Jie Li:** Investigation, Methodology, Writing – review & editing.

## Conflicts of interest

The authors declare no conflicts of interest.
